# Calculation of Deformation-Related Quantities in a Hot-Rolling Process

**DOI:** 10.3390/ma16072787

**Published:** 2023-03-30

**Authors:** Franci Vode, Simon Malej, Franc Tehovnik, Boštjan Arh, Bojan Podgornik

**Affiliations:** Institute of Metals and Technology, Lepi pot 11, 1000 Ljubljana, Slovenia; simon.malej@imt.si (S.M.); franc.tehovnik@imt.si (F.T.); bostjan.arh@imt.si (B.A.); bojan.podgornik@imt.si (B.P.)

**Keywords:** true stress vs. true strain, transfer function, average flow stress, flow-stress derivative, work done per unit deformation

## Abstract

The hot deformation of metal as a nonlinear system is mathematically described by a local linear model associated with the working conditions using a transfer function (TF) in the Laplace domain. Experimental data (true stress vs. true strain curves) are obtained using the established compressive uniaxial deformation test, where experimental conditions (strain rate and temperature) define the working conditions of the local linear TF model, which is intrinsically a function of strain. Based on the TF model, three important physical quantities of the tested metal are determined exactly: the work done per unit deformation, the average flow stress, and the flow-stress derivative with respect to the strain based on a particular TF. The exactly determined quantities, determined as a function of strain, can replace the previously used approximations in some rolling force and torque calculations.

## 1. Introduction

For accurate dimensional control of a hot-rolling process, the roll-separation force and the torque are the most important quantities for both forward production planning and real-time process control. Additionally, the roll-separation force and the torque during hot rolling are limited by the equipment of the rolling mill. Both quantities depend heavily on the nonlinear flow of the rolled metal as well as the geometrical setup. An accurate description of the flow rule is, therefore, of paramount importance.

Published reviews on the roll-separation force and the torque can be found in [1] and more extensively in [2]. The main problem when solving the roll-separation force and the torque is to calculate the roll-pressure distribution along the angle on the roll, where the rolled steel is in contact. The names of the variables follow the notation of [3]. Simple models for calculating the roll-separation force and the torque need one or more of the following quantities: average stress $\sigma_{FM}$, strain-dependent flow rule σ(ε), strain rate for a given strain $\dot{\varepsilon }\left(\varepsilon \right)$, derivative of the strain-dependent flow rule with respect to angle ∅, dσ/d∅ (or with respect to *x*, depending on the geometry defined for the normal roll-pressure distribution), where ∅ is the local angle at the roll/slab contact. The derivative of flow dependent with respect to angle ∅ is obtained using the chain rule of differentiation through the intermediate variable strain ε and is

(1) dσ/d∅=dσ/dϵ∗dϵ/d∅.

Simple models employ explicit equations for the average pressure per unit width of Schey and Sims [2,4], which require $\sigma_{FM}$ as a multiplier. A more complex model developed firstly by Karman [5] (Karman equation) and later improved by Orowan [6] derives the roll-pressure distribution by solving a differential equation for the equilibrium of forces acting on a deformed slab. A crucial improvement to Orowan’s solution of Karman’s is the consideration of the mixed boundary condition at the roll/slab interface τ=μs and τ=k, where μs>k, with *s* being the local pressure, μ is the coefficient of friction, and *k* is the yield/flow stress of the rolled slab. In the solution procedure, a general flow-stress distribution *k*(∅) along the contact arc and its derivative and the general coefficient of friction along the contact arc are considered. Orowan’s method includes a graphical solution of the differential equation. A simplified Orowan’s model is obtained in [7,8] by eliminating the yield-stress derivative and reformulating Orowan’s method just for a numerical solution. Freshwater employs Swift’s stress-strain hardening law *Y = Y*_0_ (1 + *B*$\bar{\epsilon }$)*^n^*. Alexander [3,8] numerically solved Orowan’s equation for an arbitrary variable-yield stress along the contact arc and also considered the flow-stress approximation based on Swift’s stress-strain hardening law. Bland and Ford [9] generally considered the strain-dependent flow stress and evaluated the error introduced by replacing it with the average flow stress $\sigma_{FM}$. There are also other strain-dependent flow models describing, specifically, strain hardening which is the research by Holomon, Ludwik, and Voce [10]. Other flow models describe only the strain softening or mixed strain hardening/softening above a certain strain [10]. Note that the above-mentioned models are approximative. On the other hand, an exact strain-dependent flow rule σ(ε) enables an exact description of the strain-dependent flow rule, regardless of the strain hardening, softening, or any mixed modes. Thus, using a single function (TF) that accurately describes the flow stress as a function of strain can, in some situations, be an advantage. Additionally, using the same TF for an exact calculation of other statistical values, such as the average flow stress $\sigma_{FM}$, brings additional benefits.

Two similar models that are able to analytically calculate the roll-separation force are based on the total energy minimization principle, employing a constant flow-stress formulation [11,12].

Another important variable during hot rolling is the rolled metal’s temperature, where any change during hot rolling greatly depends on the energy introduced through the deformation work. The temperature increase caused by the deformation work can be estimated by dividing $\int \sigma \left(\varepsilon \right)d\varepsilon$ by the specific heat and the density of the deformed material [2,13,14].

To summarize the rolling force and torque problems, the strain-dependent flow rule, the average flow stress, the flow-stress derivative with respect to angle ∅, and the work done per unit deformation are quantities that appear in many (mostly simple) rolling force and torque calculation methods, but their calculations are mostly simplified. Here we show how these quantities can be accurately calculated.

**Figure 1:**
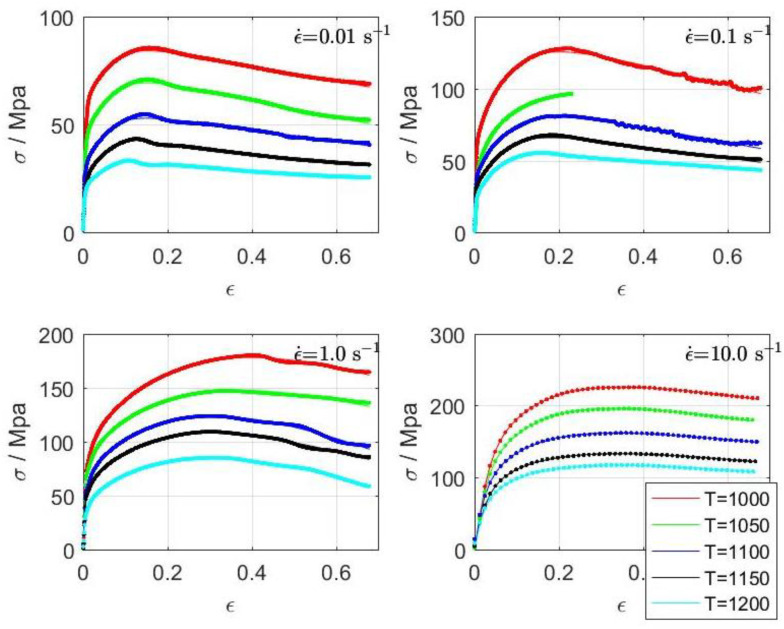
Tightly matching measured and calculated true stress vs. true strain curves for temperatures (1000, 1050, 1100, 1150, and 1200 °C) and strain rates (0.01, 0.1, 1, and 10 s^−1^).

In [15], it was shown that for a uniaxial compressive hot-deformation experiment, the stress response under a constant strain rate could be very accurately modeled (note the hardly visible difference between the measured and calculated stresses–Figure 1) using a third-order TF and slightly less accurately using a 2nd-order TF. In a configuration with the strain rate as input and the stress $\sigma \left(\varepsilon \right)=h\left(\dot{\varepsilon }\left(\varepsilon \right)\right)$ as output, optimization as a method for determining the transfer-function coefficients converges more easily compared to the strain-for-input approach. This is due to the coefficients for various strain rates and temperatures being closer to each other. Such a configuration reduces optimization efforts but is not as general as the strain-for-input configuration $\sigma \left(\varepsilon \right)=g(\varepsilon \left(\varepsilon \right))$ [15]. However, in both cases, the Laplace transformation is used along the strain ε axis.

The configuration of strain as input and stress as output along the time axis $\sigma \left(t\right)=h(\varepsilon \left(t\right))$ is more general and offers conditional swapping between the time and the deformation. Under a constant strain-rate assumption, $\sigma \left(t\right)=h\left(\varepsilon \left(t\right)\right)$ can be transformed in the Laplace domain into a form describing it along the strain axis; $\sigma \left(\varepsilon \right)=f\left(\varepsilon \left(\varepsilon \right)\right)$, as well as back in the time domain. Mathematically, in the Laplace domain, this transform is a division of TF zeros and poles by a constant (in this case, the constant is the strain rate $\dot{\varepsilon }$). Swapping the *t-*ε space is essential for later calculations presented here.

In the paper, the presented approach follows that of a local linear model for a description of the nonlinear system around the working point or equilibrium [16,17,18,19,20], where the TF model is dynamic in its nature and should therefore be clearly different to any kind static system [21].

In the article, we demonstrate that by describing the true stress vs. true strain relation using a transfer function, three important quantities in the field of hot deformation for metals can be calculated as a continuous function of time (or deformation): the work done per unit deformation, the average flow stress and the flow-stress derivative with respect to strain. Furthermore, an accurate true stress vs. true strain relation described by the TF as an intrinsic function of strain might, in some situations, accurately replace multiple separate approximative models describing the strain hardening and/or the softening of the metal as a function of strain.

## 2. Materials and Methods

### 2.1. Experimental

The material used in this investigation was spring steel grade 51CrV4, with the chemical composition given in Table 1. The steel was produced in a laboratory-scale induction furnace (capacity 20 l, max. power on inductor 100 kW), cast into ingots of 60 × 60 mm cross-section, hot rolled to a bar thickness of 22 mm in seven passes using a Schmitz two high rolling mill with a 95 kW electrical motor. The diameter of the rolls and the rotation speed were 296 mm and 27 min^−1^, respectively. From the resulting bars, cylindrical samples of 5 × 10 mm were machined, with the cylinder axis in the rolling direction. A deformational dilatometer apparatus, TA Instruments 805A/D (TA Instruments, New Castle, PA, USA), was used for the determination of the true stress vs. true strain curves, shown in Figure 1. The compression tests were performed with a constant strain rate to obtain the material behavior at a specified strain rate. Outside of compressive tests, tensile and torsion tests were used [2]. The experiments were performed at different temperatures (1000, 1050, 1100, 1150, and 1200 °C) and strain rates (0.01, 0.1, 1, and 10 s^−1^), with the original results being presented in earlier published work [15]. Standard, non-lubricated 0.1 mm thick Mo plates were placed on the sample contact surfaces. The experimentally obtained true stress vs. true strain curves were not friction corrected. Numerical optimizations were performed in Octave [22], while some other computations used MATLAB [23].

### 2.2. Transfer Function and Differential Equation

A transfer function (TF) of the third order is defined as a quotient of the Laplace transform [24] of the output signal and the input signal, where the Laplace transform of both output and input signals is a polynomial form of the power of *s,* as defined in Equation (2):

(2) $$\frac{L\left(y(t)\right)}{L\left(x(t)\right)}=\frac{Y(s)}{X(s)}=G\left(s\right)=\frac{a_{3}s^{3}+a_{2}s^{2}+a_{1}s+a_{0}}{b_{3}s^{3}+b_{2}s^{2}+b_{1}s+b_{0}}$$

where *s* is the Laplace parameter, *a*_3_*… a*_0_ are coefficients of the numerator polynomial, *b*_3_*… b*_0_ are coefficients of the denominator polynomial, and TF (s) is defined in the Laplace domain. The independent variable in the Laplace transform is usually time. For a visualization, Equation (2) is transformed in the time domain by recalling the relation

$$\frac{df}{dt}\left(t\right)\overset{{L,L}^{-1}}{\Leftrightarrow }sF\left(s\right)-f(0)$$

The relation for the n-th order time derivatives and supposing zero initial conditions $f\left(0\right)=0$ yields

(3) $$b_{3}\dddot{y}+b_{3}\ddot{y}+b_{3}\dot{y}+b_{o}=b_{3}\dddot{x}+b_{3}\ddot{x}+b_{3}\dot{x}+a_{o}$$

where $\dot{x}$ is the first-time derivative and $\ddot{x}$ is the second-time derivative of *x*(*t*), etc. Equation (3) is a linear, ordinary differential equation of the third order. The second-order *G*(*s*) (*a*_3_ = 0 and *b*_3_ = 0) similarly leads to a second-order ordinary differential equation. TFs in the Laplace domain of form (2) are, therefore, in the time domain, equivalent to the linear ordinary differential Equation (3).

### 2.3. Replacing Time with Strain in the Laplace Transform

The transformation of the time-based *G*(*s*) into the strain-based *G*(*s*) can be performed under a constant strain rate assumption, under which the relation between the strain and the stress is $\varepsilon =\dot{\varepsilon }t$.

The following pair between the time domain and the Laplace domain function holds [24]:

(4) $$f\left(ct\right)\overset{{L,L}^{-1}}{\Leftrightarrow }\frac{1}{c}F\left(\frac{s}{c}\right)$$

where *c* is a constant. Using the strain rate as *c* = $\dot{\varepsilon }$ and the relation $\varepsilon =\dot{\varepsilon }t$ leads to

(5) $$f\left(\varepsilon \right)\overset{{L,L}^{-1}}{\Leftrightarrow }\frac{1}{\dot{\varepsilon }}F\left(\frac{s}{\dot{\varepsilon }}\right)$$

Strain-based or time-based defined TFs are required, depending on the requirements. The latter form of TF (strain-based) in the Laplace domain enables a calculation of the average stress per unit width during hot rolling.

### 2.4. Identification of G(s) Parameters by Optimization

Equation (2) can be used to identify the parameters of *G*(*s*). The usually measured data of the uniaxial compressive or tensile stress vs. strain relation provide both the measured stress and strain in the time domain. Let us provide some details about the identification of *G*(*s*) using Equation (2). Identification is based on time-domain measurements of the stress $\sigma \left(t\right)$ and strain $\varepsilon \left(t\right).$ According to Equation (2), we need to transform the time-based measurements of stress $\sigma \left(t\right)$ and strain $\varepsilon \left(t\right)$ into Laplace-domain measurements of the stress $\sigma \left(s\right)$ and strain $\varepsilon \left(s\right).$ This is, however, not analytically possible. A more feasible path is to form an optimization problem, where six parameters {*a*_3_, *a*_2_, *a*_1_, *a*_0_, *b*_3_, *b*_2_, *b*_1_, *b*_0_} are determined so that the difference between the measured stress $\sigma_{m}\left(t\right)$ and the calculated stress $\sigma_{c}\left(t\right)$ along the whole $\sigma \left(t\right)$ curve is minimized. The calculated $\sigma_{c}\left(t\right)$ stress can be defined as $\sigma_{c}\left(t\right)=L^{-1}\{G\left(s\right)L(\varepsilon_{m}\left(t\right))$.

At this point, the established numerical methods for the calculation of the Laplace and the inverse-Laplace transform can be used for a single-function calculation of the $\sigma_{c}\left(t\right)$ curve according to selected set of parameters {*a*_3_, *a*_2_, *a*_1_, *a*_0_, *b*_3_, *b*_2_, *b*_1_, *b*_0_}. The procedure should be performed for each call during the optimization with a varying set of parameters {*a*_3_, *a*_2_, *a*_1_, *a*_0_, *b*_3_, *b*_2_, *b*_1_, *b*_0_}. The optimization procedure is more problematic. In this case, the ‘lsqnonlin’ optimization function in Octave is used. Several initialization options offered by ‘lsqnonlin’ are applied, while neither the upper nor the lower bounds are used. In most cases, no stable convergence towards the measured stress curve can be observed, thus requiring many trials with various settings and initial optimization points. When the TF parameters are determined for one temperature vs. strain rate condition ($T,\dot{\varepsilon }$), the TF parameters of ($T,\dot{\varepsilon }$) might be a very good initial value of the TF parameters for neighboring pairs ($T_{1},{\dot{\varepsilon }}_{1}$), although unfortunately, it may not necessarily be so.

When $\sigma_{m}\left(t\right)$-$\sigma_{c}\left(t\right)$ is sufficiently low along the whole curve, the optimization converges towards an approximate solution of the kernel *G*(*s*) in the Laplace domain.

Excitation with a unit step function or ramp function, as in this case of a linearly increasing deformation ε, is among the less desired [20,21,25], while among the most desirable excitations are waveforms covering a wider frequency spectrum, e.g., a pseudo-random binary signal. Bearing in mind the limitation of the monotonic deformation process, as used in this case, considering the strain as the input and the strain-rate sensitivity/dependency of the system, very prohibitive options remain regarding the system-excitation signal type. These are only monotonically increasing deformations with variable inclinations combined with intermediate pauses in the deformations being possible.

## 3. Results

Once the *G*(*s*) parameters are identified as described above, the stress curve $\sigma_{c}\left(t\right)$ can be calculated and compared to the experimentally obtained $\sigma_{m}\left(t\right)$ stress curve. This comparison for a set of different temperatures and strain rates ($T,\dot{\varepsilon }$) is shown in Figure 1. Note the tightly matching measured and calculated strains. The obtained TFs–*G*(*s*) for each temperature and strain rate are used for the calculations shown below.

### 3.1. Calculation of Average Stress $\sigma_{FM}$ during Hot Rolling and Work Done per Unit Deformation

The work done per unit of deformation is a calculation of integral [26]:

(6) $$w(\varepsilon )=\int_{0}^{\varepsilon_{max}}\sigma (\varepsilon )d\varepsilon$$

**Figure 2:**
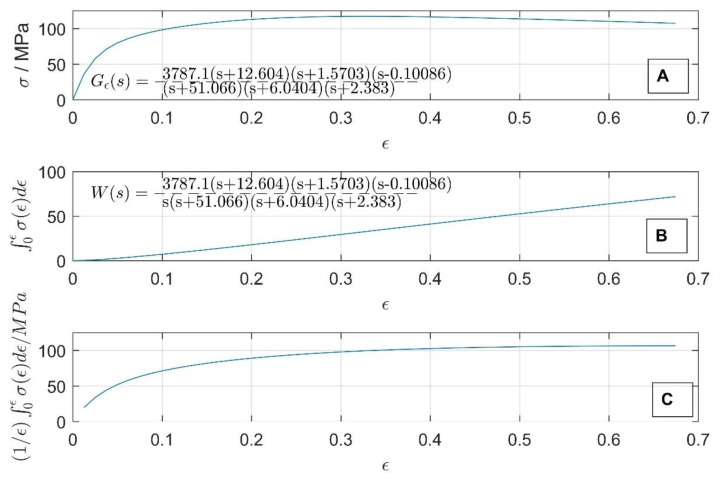
True stress vs. true strain curve $G_{\varepsilon }(\varepsilon )$ (**A**) for 1000 °C and a strain rate 0.01 s^−1^, work done per unit deformation (**B**) and calculated average flow stress $\sigma_{FM}$ (**C**).

Equation (6) in the Laplace domain reads as $w\left(s\right)=G_{\varepsilon }\left(s\right)1/s$ and its solution is obtained by performing an inverse Laplace of $w(\varepsilon )=L^{-1}\left(G_{\varepsilon }*1/s\right)$. The values of *w*(*ε*) and its Laplace domain equivalent *W*(*s*) are shown in Figure 2B, with the original flow-stress curve and its TF $G_{\varepsilon }(s)$ in the Laplace domain shown in Figure 2A.

Similarly, the average flow stress $\sigma_{FM}$ is defined as

(7) $$\sigma_{FM}\left(\varepsilon \right)=\frac{1}{\varepsilon_{max}}\int_{0}^{\varepsilon_{max}}\sigma \left(\varepsilon \right)d\varepsilon$$

where $\varepsilon_{max}=ln(h_{entry}/h_{exit})$ is the maximum true strain defined by the entry and exit rolling piece height during a hot-rolling pass. However, the average flow stress cannot be easily defined in the Laplace domain, since TF $G_{\varepsilon }\left(s\right)$ is obtained by performing a Laplace transform along the strain axis, with the equation for $\sigma_{FM}$ (7) following a time-domain Laplace transform pair: $1/t*f(t)\overset{L,L^{-1}}{↔}\int_{s}^{\infty }F(u)du$. To obtain a Laplace-domain equivalent of $\sigma_{FM}(\varepsilon )$, we should calculate the integral of the expression $G_{\varepsilon }\left(s\right)$ in the Laplace domain. A way around this is to use values of w(ε) (6) in the strain domain and divide the temporary values of $w\left(\varepsilon \right)$ via the temporary deformation (ε). In this way, the calculated values of $\sigma_{FM}(\varepsilon )$ for the values of $0<\varepsilon_{max}<0.7$ are shown in Figure 2C.

### 3.2. Calculation of Flow Stress Derivative

**Figure 3:**
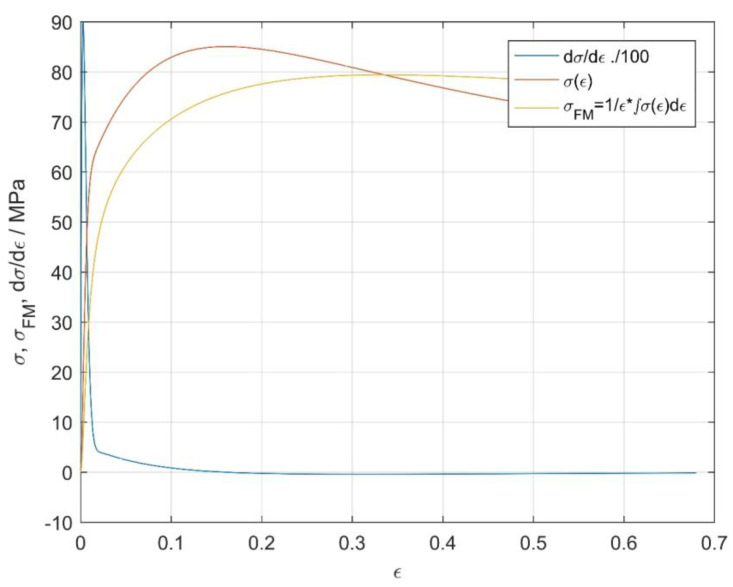
Flow curve $G^{\prime }(s)\overset{L,L^{-1}}{↔}\sigma (\epsilon )$ at (1000 °C, 0.01 s^−1^), flow stress derivative with respect to strain dσ/dϵ(ϵ) and average flow stress $\sigma_{FM}\left(\epsilon \right)$. All three quantities are determined along whole deformation range as continuous functions of strain ϵ based on *G*′(*s*).

The expression for the flow-stress derivative dσ/dϵ in the Laplace domain using the same order of numerator and denominator in the TF leads to a non-causal system, although it can be simply expressed in the Laplace *s*-domain by TF multiplication with *s* and is useless for a real-time application. The flow-stress derivative in the Laplace domain can, however, be evaluated in real time (causal) for TFs, where the order of the numerator polynomial is at least one less than the order of the denominator polynomial. Fortunately, the stress-strain relation can be expressed with similar accuracy using TF with a 4th-order denominator and a 3rd-order numerator (thereafter assigned as G’(s)), as shown in Figure 3. The *G*′(*s*) presented in Figure 3 are identified using experimental data for 1000 °C and a strain rate = 0.01 s^−^^1^ and represent *G*′(*s*) (σ(ϵ)), the derivative of *G*′(*s*) (dσ/dϵ), and the curve of mean flow stress as a function of strain $\sigma_{FM}$. Based on distinct *G*′(*s*), we can obtain an exact and causal expression of (1) the derivative of the flow stress, (2) the average flow stress $\sigma_{FM}$, and (3) the work done per unit deformation based on a single *G*′(*s*) expression and as continuous functions along the whole deformation range. The same procedure can be used for the determination of these quantities at the remaining strain rates and temperatures.

## 4. Discussion

Maybe the most important property of the TFs application for a description of the true stress vs. true strain relation is that the notation opens the path for exact calculations of (a) the strain-dependent flow stress, (b) the average flow stress, (c) the flow-stress derivative with respect to angle ∅, and the work done per unit deformation.

Some additional notification regarding the expression for the flow-stress derivative dσ/dϵ using TFs. The inversion of *G*(*s*) is possible only if the order of the numerator and the denominator in *G*(*s*) is the same. The expression of the flow-stress derivative leading to a causal expression requires the order of the numerator to be at least one less than the denominator in *G*(*s*). In other words, for invertible *G*(*s*), the flow-stress derivative cannot be expressed as a causal system. While for *G*′(*s*), for which the flow-stress derivative can be expressed, inversion is not possible (as it leads to a non-causal system). The same *G*(*s*) is either not invertible or leads to a non-causal expression of the flow-stress derivative. Two distinctive *G*(*s*) and *G*′(*s*), where the first is reversible and the second leads to a causal flow-stress derivative, seem to coexist for the same stress-strain relation. Currently, it is unclear whether some transformation or algebraic operation exists to convert *G*(*s*) to *G*′(*s*).

The TF formulation of the true stress vs. true strain relation may appear to some readers as a complicated formulation and/or computationally demanding formulation. Fortunately, the TF formulation of dynamical systems is in daily use in industrial implementations of programmable logical controllers (PLCs) and other control devices [15], where PLC scan times are typically below 100 ms. The computation time for the calculation of the TF response on PCs and other more computationally powerful platforms is accordingly shorter. So, applications of the stress-strain description using TFs could, due to computational bounds, be used in various time-non-critical applications, while the use of TFs-based stress-strain relations in time-critical high-speed loops of automatic gauge control might be limited or inappropriate [8]. The use of TFs for various offline calculations for a description of the stress-strain relation for rolling pass schedule optimization tasks [27] with underlying roll force and torque calculations is a minor computational burden compared to all remaining calculations needed for a determination of roll force and torque [3,5,6].

Additional remarks regarding the locality of TF models: nonlinear system identification by local linear models is a viable and most frequently used method for nonlinear system description in the vicinity of the working conditions [20]. An individual compressive deformation test performed under a constant strain rate and temperature, as used in this article, is deformed to deformation, e.g., $\varepsilon_{max}$ = 0.7. An equivalent test under constant stress (creep) resulting in only $\varepsilon_{max}$ = 0.1 leads to entirely different TFs. Thus, the system is inherently nonlinear. For accurate model predictions, the proposed TF model should be used around working conditions.

## 5. Conclusions

An accurate description of hot-compressive true stress vs. true strain curves using TFs as a function of strain enables an accurate description of a metal’s response to deformation/strain, having a strain-hardening, softening, or mixed-mode response to deformation/strain. Besides this, three additional quantities commonly used in the calculation of the roll-separation force and torque can be exactly calculated as a function of deformation/strain: (1) the work done per unit deformation, (2) the average flow stress, and (3) the flow-stress derivative based on distinct *G*′(*s*).

## Figures and Tables

**Table 1:** Chemical composition of 51CrV4 steel in wt.%.

C	Si	Mn	Cr	V
0.5	0.30	0.95	1.00	0.15

## Data Availability

Ref. [28] is cited in Supplementary Materials.

## Supplementary Materials

The following supporting information can be downloaded at: https://data.mendeley.com/datasets/xm85449wg5/1. Experimental true stress vs. true strain measured data for all temperatures and strain rates are provided on Mendeley data. Optimization scripts for the identification of *G*(*s*) and *G*′(*s*), including simulation and figure generation of the obtained TFs.
